# Development and external validation of a prostate health index-based nomogram for predicting prostate cancer

**DOI:** 10.1038/srep15341

**Published:** 2015-10-16

**Authors:** Yao Zhu, Cheng-Tao Han, Gui-Ming Zhang, Fang Liu, Qiang Ding, Jian-Feng Xu, Adriana C. Vidal, Stephen J. Freedland, Chi-Fai Ng, Ding-Wei Ye

**Affiliations:** 1 Department of Urology, Fudan University Shanghai Cancer Center, Shanghai, P.R. China; 2 Department of Oncology, Shanghai Medical College, Fudan University, Shanghai, P.R. China; 3 State Key Laboratory of Genetic Engineering, School of Life Sciences, Fudan University, Shanghai, P.R. China; 4 Fudan Institute of Urology, Huashan Hospital, Fudan University, Shanghai, P.R. China; 5 Department of Urology, Huashan Hospital, Fudan University, Shanghai, P.R. China; 6 Center for Genetic Epidemiology, School of Life Sciences, Fudan University, Shanghai, P.R. China; 7 Department of Surgery, Center for Integrated Research on Cancer and Lifestyle, Samuel Oschin Comprehensive Cancer Institute, Cedars Sinai Medical Center, Los Angeles, CA; 8 SH Ho Urology Center, Department of Surgery, The Chinese University of Hong Kong, Shatin, NT, Hong Kong SAR, P.R. China

## Abstract

To develop and externally validate a prostate health index (PHI)-based nomogram for predicting the presence of prostate cancer (PCa) at biopsy in Chinese men with prostate-specific antigen 4–10 ng/mL and normal digital rectal examination (DRE). 347 men were recruited from two hospitals between 2012 and 2014 to develop a PHI-based nomogram to predict PCa. To validate these results, we used a separate cohort of 230 men recruited at another center between 2008 and 2013. Receiver operator curves (ROC) were used to assess the ability to predict PCa. A nomogram was derived from the multivariable logistic regression model and its accuracy was assessed by the area under the ROC (AUC). PHI achieved the highest AUC of 0.839 in the development cohort compared to the other predictors (p < 0.001). Including age and prostate volume, a PHI-based nomogram was constructed and rendered an AUC of 0.877 (95% CI 0.813–0.938). The AUC of the nomogram in the validation cohort was 0.786 (95% CI 0.678–0.894). In clinical effectiveness analyses, the PHI-based nomogram reduced unnecessary biopsies from 42.6% to 27% using a 5% threshold risk of PCa to avoid biopsy with no increase in the number of missed cases relative to conventional biopsy decision.

Prostate cancer (PCa) has been an emerging threat in urban areas of the People’s Republic of China over the last decade^1^. The standardized incidence of PCa in male Shanghai citizens increased from 5.6 per 100,000 in 1999 to 12.96 per 100,000 in 2009. A similar trend was observed in Hong Kong where the incidence rate rose from 16.5 per 100,000 in 1999 to 28.6 per 100,000 in 2009^1^. In both cities, PCa ranked as the fifth most common male malignancy. This increase, albeit multifactorial, can be partly explained by the widespread adoption of the serum prostate-specific antigen (PSA) test in developed People’s Republic of China^2^. In a recent report of radical prostatectomy patients, 64.3% were diagnosed based on increased PSA without symptoms^3^, while this rate was only 10% in 2005^4^.

The performance of serum PSA test for PCa was previously tested in Chinese men and showed limited specificity^5^. Specifically, among men with PSAs of 4–10 ng/mL, only 19.4% had cancer on biopsy. This cancer detection rate is relatively low compared with that of Western men (36%–56%^6^), and indicates a high prevalence of unnecessary biopsies. Consequently, clinical decisions relying on PSA value alone will certainly continue to generate significant negative biopsies. To preserve the benefit of early detection while reducing overdiagnoses, several tools have been developed to increase the specificity of the PSA test, including the ratio of free-to-total PSA (%fPSA) and PSA density (PSAD). Unfortunately, %fPSA and PSAD yield a moderate discriminative ability with an area under the receiver operator curve (AUC) of less than 0.65^7^. Therefore, new tools for this unmet need are required to further improve the specificity of PCa diagnosis and to aid in clinical decision-making.

The PSA isoform, [–2]proPSA (p2PSA) is one of the most attractive approaches to overcome the abovementioned challenge^8^. Its derivative, the prostate health index (PHI), was approved by the US Food and Drug Administration for detection of PCa in men with a PSA level of 4–10 ng/mL and normal digital rectal examination (DRE). Na *et al*. evaluated PHI in Chinese patients with PSA 2–10 ng/mL and found PHI as single predictor showed an AUC of 0.73, which was significantly better than tPSA alone (0.53)^9^. The superior performance of PHI over currently used criteria was also reported by Ng *et al*.^7^. They reported a better predictive value for PHI to detect PCa at diagnosis (AUC = 0.781), compared with predictive value of tPSA (AUC = 0.547), %fPSA (AUC = 0.572), and PSAD (AUC = 0.634). However these studies did not adjust for confounders such as patient age and prostate volume. An alternative approach to increase the performance characteristics of PHI testing for PCa diagnosis is to build a multifactorial prediction model considering PSA and other risk factors^10^. Utsumi *et al*. evaluated several free and total PSA-based nomograms in Japanese patients with a PSA of 4–10 ng/mL regardless of DRE findings. The authors found a maximum AUC of 0.747 with the five assessed nomograms^11^. Furthermore, the authors showed those nomograms seemed to provide more precise risk-analysis information for individual Japanese patients than Western nomograms^11^. Therefore, incorporating PHI in a multifactorial model may be a promising solution for accurate PCa risk estimation. Although a nomogram was built and validated on men of European descent^12^, similar tools are unavailable for Chinese men.

The objective of the current study was to construct a PHI-based nomogram for Chinese men and test its performance in an external dataset. We selected men with a PSA of 4–10 ng/mL and a normal DRE because this group is at a high probability of going through unnecessary biopsies and thus stand the most to benefit from a good prediction model for PCa diagnosis. We hypothesized that this prediction tool may improve accurate risk estimation and, most importantly, aid in clinical practice.

## Results

### Descriptive characteristics of study cohorts

In the development cohort, 347 men fulfilled the inclusion criteria. For the validation cohort, a total of 230 patients were recruited. The baseline characteristics of the two cohorts are shown in Table 1. Most of the enrolled men were aged between 60 and 70 years, although the development cohort was slightly younger (p = 0.011). The distribution of prostate volume, tPSA, and p2PSA was similar between the two cohorts (all p ≥ 0.19). PHI and %p2PSA were significantly lower in the validation cohort (p < 0.001), as well as the incidence of PCa (p = 0.052) and high-grade disease (p = 0.001).

### Development and internal validation of the nomogram for predicting prostate cancer

**Figure 1:**
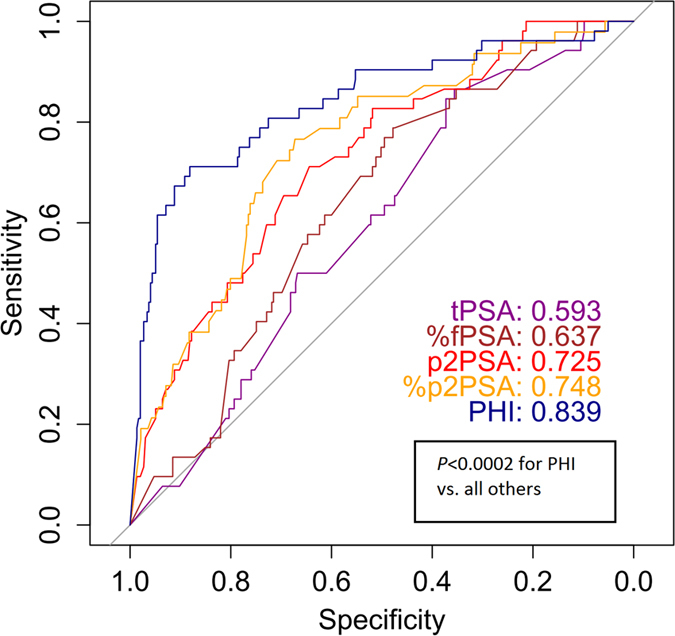
Receiver operating characteristic curve analyses of predictors for prostate cancer in the development cohort. Abbreviations: PSA = prostate specific antigen, tPSA = total PSA, fPSA = free PSA, p2PSA = [−2]proPSA, PHI = prostate health index.

ROC analyses were performed to assess the discriminative ability of PSA-derived predictors in the development cohort. As shown in Fig. 1, PHI achieved the highest AUC of 0.839 (95% confidence interval [CI]: 0.771–0.907) and was significantly better than other predictors (*P *< 0.0002 in all paired comparisons). The benefit of PHI was most pronounced for higher sensitivity without loss of specificity. This phenomenon indicates that PHI is associated with lower false positive predictions.

**Figure 2:**
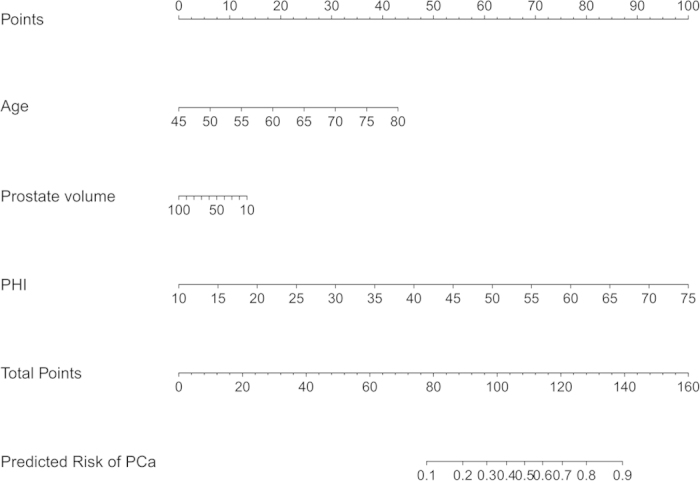
PHI-nomogram predicting the probability of prostate cancer in Chinese men with PSA ≤10 ng/mL and normal digital rectal examination. Instructions: To obtain the nomogram-predicted probability, locate patient values on each axis. Draw a vertical line to the point axis to determine how many points are attributed for each variable value. Sum the points for all variables. Locate the sum on the total point line to assess the individual probability of prostate cancer at biopsy. Abbreviations: PHI = prostate health index, PCa = prostate cancer.

Multivariable analysis was performed to construct a prediction model for estimating PCa risk. As expected, age and PHI were strongly associated with positive biopsy (Table 2). For instance, a 1-unit increase in PHI was associated with an 11.6% increase in PCa risk at biopsy. The AUC of the multivariate model was 0.877 (range, 0.813–0.938) and a nomogram was graphically depicted based on these results (Fig. 2).

The PHI-nomogram was internally tested in the development cohort. Using a 200 bootstrap resampled dataset, the nomogram’s corrected AUC was 0.872, with only a slight decrease (−0.005) in discriminative ability. The calibration plot showed good correlation between predicted and actual probability (Supplementary Figure S1). The Hosmer–Lemeshow test also indicates a good fit for the model calibration (*P *= 0.236).

### External validation of the nomogram and assessment of clinical usefulness

The PHI-nomogram was assessed for external validation by measuring discrimination, calibration, and clinical usefulness in the validation cohort. In this second separate cohort, the AUC of the nomogram was 0.786 (range, 0.678–0.894). We evaluated the influence of case-mix on the predictive performance of the nomogram. Using the validation cohort, a multifactorial model was built based on age, prostate volume, and PHI, and resulted in an AUC of 0.788 (range, 0.684–0.892). Therefore, the discriminative ability of the PHI-nomogram on the external dataset was nearly the same as the maximum performance achieved using these predictors.

**Figure 3:**
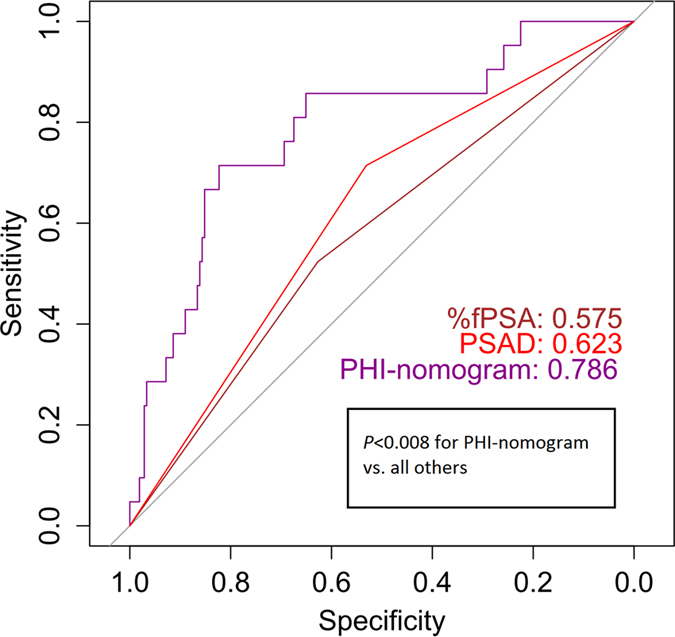
Receiver operating characteristic curve analyses of PHI-nomogram and traditional criteria in the validation cohort. Instructions: %fPSA means biopsy when %fPSA ≤0.16, PSAD means biopsy when PSA density ≥ 0.15. Abbreviations: PSA = prostate specific antigen, fPSA = free PSA, PSAD = PSA density, PHI = prostate health index.

**Figure 4:**
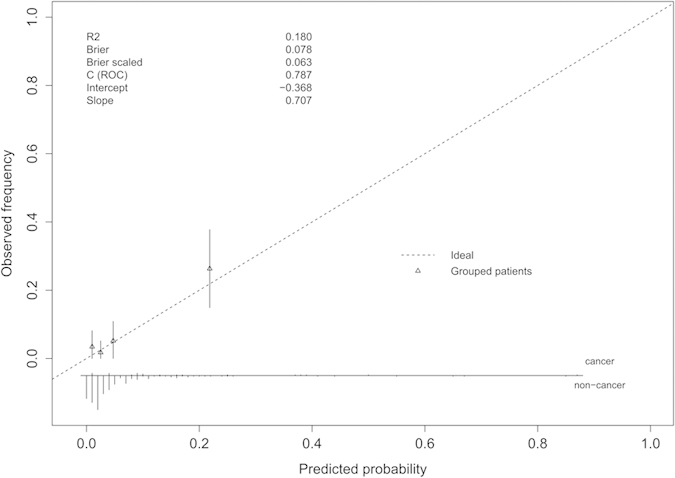
Calibration plot, where the x-axis represents the predicted probability and the y-axis represents the observed fraction of prostate cancer in the validation cohort. Instructions: The 45° dashed line represents ideal predictions, the triangle represents patient groups, the histogram at the bottom of the plots shows the distribution of outcomes, and the statistics at the upper left shows the model performance. The plot **visualizes** the proportion of patients falling within various predicted ranges when the nomogram is applied.

Using ROC analyses, the performance of the PHI-nomogram was significantly better than the currently used criteria (%fPSA and PSAD; *P *< 0.008 in all paired comparisons; Fig. 3). As shown in the calibration plot (Fig. 4), predicted risk generally matched the observed frequency. The Hosmer–Lemeshow test showed consistent results (*P *= 0.115). Decision curve analysis confirmed the highest net benefit of the PHI-nomogram over current criteria in a broad spectrum of PCa risk (from 4% to nearly 40%) (Supplementary Figure S2).

Finally, we simulated clinical decision making by calculating the consequences of applying different criteria in the validation cohort. As shown in Table 3, PSAD biopsy criteria resulted in unnecessary biopsies in 42.6% of cases and missed 28.6% of cancer cases. Using the PHI-nomogram, we were able to significantly reduce the number of unnecessary biopsies. For example, at the cutoff of 5%, unnecessary biopsies were reduced to 27% without missing any additional cancer cases.

## Discussion

PCa diagnosis is a highly controversial debate in urology clinics. The current strategy for urologist in clinical practice includes being well informed of the risk of disease probability and benefit/harm of interventions. Therefore, synthesizing a patient’s complex characteristics into a comprehensive, objective prediction of outcome is of great interest. Several prediction models have been developed to provide a precise estimation of risk. Recently, new tests based on PSA, such as PHI, have been employed in prediction models and have been shown to significantly increase predictive accuracy of PCa diagnosis in men of European descent^12^. However, the use of this new test in Chinese men has not been well-studied. Although two reports from Chinese men showed PHI correlated well with biopsy outcomes^7^,^9^, no clinically useful model has been developed and externally validated. We found that a PHI-based nomogram was best to predict PCa at biopsy in Chinese men. The nomogram generated in the current study may fill an important gap in current clinical practice in China by not only providing a validated tool but also by helping reduce unnecessary PCa biopsies. Further validation is warranted to better understand the patient population for which this nomogram performs the best.

The superior diagnostic value of PHI over current criteria was evidenced in the development cohort, in concordance with previous reports^7^,^9^. Only PHI achieved an AUC over 0.8 (0.839), compared to tPSA, %fPSA, p2PSA and %p2PSA. This was true as well after utilizing a model adjusted for age and prostate volume. In the validation cohort, the PHI nomogram was significantly superior to the current criteria of %fPSA and PSAD, which were recommended by the Chinese urological guidelines to be used in patients with PSA 4–10 ng/mL^13^. The nomogram presented herein is a practical tool developed to offer **individualized** prediction of biopsy outcome. Different from most previous studies, our model was externally validated to show the robustness of risk estimation. The validated performance of our nomogram was comparable with a European study (AUC = 0.752)^12^. Most importantly, the nomogram achieved nearly maximum AUC in the validation cohort, suggesting the correctness of regression coefficients. Taken together, the PHI-nomogram was able to achieve a predictive accuracy of more than 0.75 in the validation cohort and can be used as a new reference standard for further markers.

Our study has several strengths. First, the study population included those with PSAs of 4–10 ng/mL and normal DRE. These patients fall into the so-called “gray-zone” and are the mostly likely to undergo biopsy needlessly. According to the literature, few studies have investigated such a population in Chinese men. Owing to the widespread use of the PSA test, Chinese men were more frequently referred for biopsy under these conditions^3^. The nomogram, therefore, provides a timely tool with better accuracy than current tools recommended by the Chinese urological guideline^13^.

Second, our study included a development and an independent validation cohort. PCa ranks as the third and fifth most prevalent cancer in Shanghai and Hong Kong, respectively^1^. Using cohorts in those two developed areas of the People’s Republic of China was ideal for constructing the prediction model because men living in those cities share similar inherent characteristics and environmental exposures that are common in urban areas. The promising performance of the nomogram in the validation cohort justifies its use in Chinese men. Future studies will test its accuracy outside of China.

Third, the nomogram not only provided **individualized** risk estimation, but also provided substantial clinical usefulness. In clinical practice, a **dichotomized** cutoff is usually set up to determine the appropriateness of a biopsy. In such situations, the nomogram was able to significantly reduce unnecessary biopsies compared with %fPSA and PSAD criteria. Because PCa incidence is relatively lower in Chinese men, compared to Western men, the challenge to reduce unnecessary biopsies remains a predominant problem.

Nevertheless, the decision to perform a prostate biopsy not only depends on PCa risk, but also on multiple factors, including the patient’s life expectancy, co-morbidity, and preference. Our nomogram provides an objective and quantifiable estimation of cancer risk and offers useful information for consultation.

Our study has several limitations. For instance, the nomogram does not include important criteria such as PCa aggressiveness. Because the number of high-grade disease were low, we had limited power to construct a robust model for predicting high-grade PCa. Using a whole-mount technique pathological analysis of men undergoing radical prostatectomy, we previously showed that 87.8% of localized PCa in Chinese men was clinically significant (pathological T stage > T2 or Gleason > 6)^3^. Second, the population was subjected to some selection biases because recruitment occurred in tertiary **centers**. Therefore, further study is required to assess whether the nomogram can be **generalized** to community screened patients. Third, our outcome was cancer on biopsy. It is well-established that cancers can be missed on biopsy resulting in less than ideal prediction. Despite this limitation, our PHI-based nomogram performed extremely well. Finally, the PHI-nomogram is only one step toward better cancer risk estimation. The additional value of other markers, such as PCA3, and other imagining techniques, such as magnetic resonance imaging, should be further assessed.

In summary, we developed and externally validated a PHI-based nomogram that accurately predicted individual PCa risk among Chinese men and if implemented in clinical practice, may help prevent unnecessary biopsies. Future studies are needed to confirm these findings and test its validity in other patient populations.

## Methods

### Study population

The development cohort was derived from consecutive patients undergoing prostate biopsy at Fudan University Shanghai Cancer Center and Fudan University Huashan hospital in Shanghai. The period for recruitment was between April 2012 and August 2014. The validation cohort was derived from patients from the Chinese University of Hong Kong in Hong Kong, between April 2008 and April 2013. The biopsy indication was the same in the two hospital-based cohorts: PSA > 4 ng/mL or abnormal DRE. At least 10 cores were taken under transrectal ultrasound guidance according to a systematic template. Specific genitourinary pathologists evaluated the biopsy findings. Serum p2PSA, tPSA, and fPSA of each patient were measured using Beckman Coulter’s DxI 800 Immunoassay system in our central laboratory. %fPSA and percentage of p2PSA (%p2PSA) were calculated as 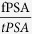 and 
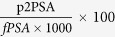
, respectively. PHI was calculated according to following formula: 
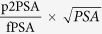
. PSAD was calculated as tPSA divided by the ultrasound measured prostate volume.

For the current analyses, the inclusion criteria were initial prostate biopsy with PSA of 4–10 ng/mL and normal DRE. The exclusion criteria included acute prostate infection, history of 5-α reductase inhibitor use, and serum sample archived for more than 3 years. The study was carried out in accordance with the ethical standards of the Helsinki Declaration II and approved by the Institution Review Board of Fudan University Shanghai Cancer Center. Written informed consent was obtained from each patient before any study-specific investigation was performed.

### Statistical Analysis

Our primary outcome was PCa diagnosis at biopsy and our exposures were serum tPSA, %fPSA, p2PSA, %p2PSA, PSAD, and PHI, were calculated, using standard formulas^14^. For comparisons of characteristics between the two cohorts, chi-squared and Kruskal–Wallis rank sum tests were used for categorical variables and continuous variables, respectively. The development and external validation of the nomogram for PCa comprised several steps^15^. First, the PSA-derived predictors with the highest discriminative ability were selected. Second, a multivariable model was constructed using the predictor and two clinical features a priori selected as key risk factors for PCa (age and prostate volume). The prediction model was graphically presented as a nomogram for clinical use. The model’s performance for discrimination and calibration was internally tested using bootstrap resamples. Third, the validity of the model was tested in an independent cohort with respect to discrimination, calibration, decision curve analyses, and simulated clinical decision. The discrimination was evaluated using AUC derived from the receiver operating characteristic (ROC) curves. The AUCs were compared by the DeLong test. Calibration was assessed both graphically and statistically using the Hosmer–Lemeshow test. Decision curve analysis quantified the net benefit of prognostic factors according to various thresholds. Simulated clinical decision calculated the probability of unnecessary biopsy and missing cancer risk according to various criteria. Because the discriminative ability of models can be influenced by differences in case mix, we calculated refitted performance for proper interpretation of validation results^16^. The model was refitted using validation samples and the AUC of the refitted model provided an upper boundary for performance.

All statistical analyses were performed using R and publicly available packages. Significance was set at *P *< 0.05.

## Additional Information

**How to cite this article**: Zhu, Y. *et al*. Development and external validation of a prostate health index-based nomogram for predicting prostate cancer. *Sci. Rep.* **5**, 15341; doi: 10.1038/srep15341 (2015).

## Supplementary Material

Supplementary Information

## Figures and Tables

**Table 1:** Descriptive characteristics of the development and validation cohorts.

**Characteristic**	**Development Cohort (Shanghai, China)**		**Validation Cohort (Hong Kong, China)**		**p-value**
N	347		230		
age (median [IQR])	64	[59.00, 70.00]	66	[61.00, 71.00]	0.011
prostate volume (median [IQR])	40	[29.90, 53.30]	43	[30.57, 58.00]	0.19
tPSA (median [IQR])	6.89	[5.00, 8.09]	6.56	[5.54, 7.92]	0.375
%fPSA (median [IQR])	0.17	[0.12, 0.25]	0.19	[0.15, 0.24]	0.034
p2PSA (median [IQR])	13	[9.00, 19.00]	12.87	[9.61, 17.10]	0.99
PHI (median [IQR])	32.71	[23.72, 43.61]	27.03	[22.54, 33.88]	<0.001
%p2PSA (median [IQR])	1.28	[0.91, 1.73]	1.08	[0.86, 1.35]	<0.001
prostate cancer (%)	52	(15.0)	21	(9.1)	0.052
high-grade cancer (%)	34	(9.8)	5	(2.2)	0.001

Instructions: High-grade cancer was defined as Gleason score ≥ 7.

Abbreviations: PSA=prostate specific antigen, tPSA = total PSA, fPSA = free PSA, p2PSA = [−2] proPSA, PHI = prostate health index, IQR = interquartile range.

**Table 2:** Multivariate logistic regression analysis of predictors for prostate cancer in the development cohort.

**Predictor**	**Odds Ratio**	**95% C.I.**	**χ**^**2**^ **statistics**	**p-value**
age	1.092	(1.041–1.145)	13.04	0.0003
prostate volume	0.989	(0.967–1.013)	0.8	0.371
PHI	1.116	(1.083–1.150)	51.36	<0.0001

Abbreviations: PHI = prostate health index, C.I. = confidence interval

**Table 3:** Reduction in unnecessary biopsy and number of cancer cases missed according to defined biopsy criteria in the validation cohort.

**Biopsy Criteria**	**Biopsy**			**Cancer**	
	**Performed (%)**	**Avoided (%)**	**Unnecessary (%)**	**Found (%)**	**Missed (%)**
All	230 (100)	0 (0)	209 (90.9)	21 (100)	0 (0)
%fPSA 0.16	89 (38.7)	141 (61.3)	78 (33.9)	11 (52.4)	10 (47.6)
PSAD ≥ 0.15	113 (49.1)	117 (50.9)	98 (42.6)	15 (71.4)	6 (28.6)
PHI-nomogram ≥ 5%	77 (33.5)	153 (66.5)	62 (27.0)	15 (71.4)	6 (28.6)
PHI-nomogram ≥ 10%	40 (17.4)	190 (82.6)	29 (12.6)	11 (52.4)	10 (47.6)
PHI-nomogram ≥ 20%	18 (7.8)	212 (92.2)	12 (5.2)	6 (28.6)	15 (71.4)
PHI-nomogram ≥ 30%	11 (4.8)	219 (95.2)	6 (2.6)	5 (23.8)	16 (76.2)

Abbreviations: fPSA = free PSA, PSAD = PSA density, PHI=prostate health index.
