# Supplementary material for: Development and external validation of a prostate health index-based nomogram for predicting prostate cancer
Source: Sci Rep. 2015 Oct 16;5:15341. doi: 10.1038/srep15341 (PMC4607975; doi:10.1038/srep15341)
Supplement: Supplementary Information [file srep15341-s1.pdf]

Development and external validation of a prostate health index-based nomogram for predicting prostate cancer

Yao Zhu¶, Cheng-Tao Han¶, Gui-Ming Zhang, Fang Liu, Qiang Ding, Jian-Feng Xu, Adriana C. Vidal, Stephen J. Freedland, Chi-Fai Ng*, Ding-Wei Ye*

¶Co-first authors

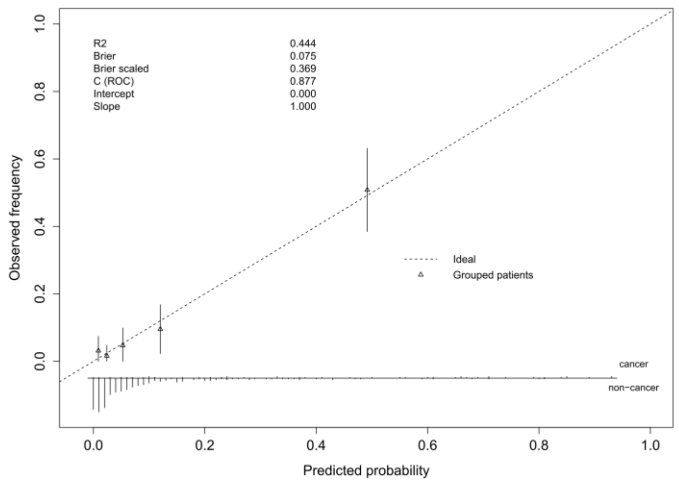

Supplementary Figure S1. Calibration plot, where the x-axis represents the predicted probability and the y-axis represents the observed fraction of prostate cancer in the development cohort.

Instructions: The 45° dashed line represents ideal predictions, the triangle represents patient groups, the histogram at the bottom of the plots shows the distribution of outcomes, and the statistics at the upper left show the model performance. The plot

visualises the proportion of patients falling within various predicted ranges when the nomogram is applied.

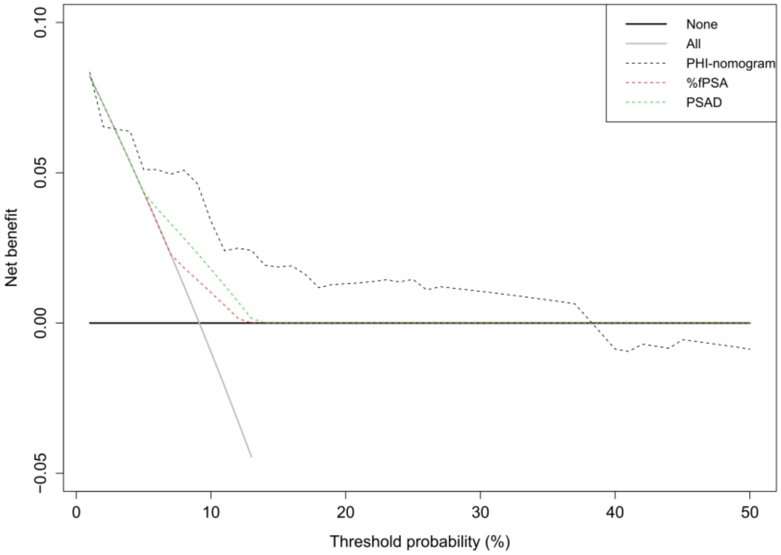

Supplementary Figure S2. Decision curve analyses of the net benefit of PHI-nomogram and traditional criteria for predicting prostate cancer in the validation cohort.

Instructions: Net benefit is plotted against threshold probabilities.
